# Hemostasis and inflammation: two of a kind?

**DOI:** 10.1186/1477-9560-7-15

**Published:** 2009-11-18

**Authors:** Peter Verhamme, Marc F Hoylaerts

**Affiliations:** 1 Center for Molecular and Vascular Biology, University of Leuven, Leuven, Belgium

## Abstract

Hemostasis is a defense mechanism to stop bleeding. Activated by vessel wall injury, it consists of intertwined activation of platelets and the coagulation cascade, tightly controlled by natural anticoagulants and the fibrinolytic system.

## Introduction

Hemostasis is a defense mechanism to stop bleeding. Activated by vessel wall injury, it consists of intertwined activation of platelets and the coagulation cascade, tightly controlled by natural anticoagulants and the fibrinolytic system [1]. Inflammation aims at restorating the integrity of damaged or threatened tissues, most frequently because of injury or infectious pathogens. The coagulation system and the innate inflammatory response share a common ancestry and are coupled via common activation pathways and feedback regulation systems. Primitive organisms as the horseshoe crab have an integrated coagulation and innate immune system [2]. More evolved species have more complex and specialized systems, but a two-way relationship between both has persisted throughout evolution: coagulation triggers inflammatory reactions and inflammation triggers the activation of the coagulation system. Extensive cross-talk between inflammation and coagulation involves cell receptor-mediated signaling, cellular interactions and the production of cell-derived microvesicles by endothelial cells, leukocytes and platelets [3].

The role of platelets in (vascular) inflammation is illustrative for this two-way relationship. After adhering to an injured vessel wall, activated platelets release cytokines, growth factors, and numerous proinflammatory mediators [4]. In addition, leukocytes are recruited to the site of vascular damage via adhered platelet-leukocyte interactions, mediated by P-selectin expressed on the activated platelet surface and its counter-receptor on leukocytes, i.e. P-selectin Glycoprotein Ligand-1 (PSGL-1) [5]. The same ligand recruits circulating microvesicles from leukocytes to the platelet surface [6], providing rapid intravascular accumulation of microvesicular tissue factor (TF) that sustains coagulation initially triggered by vascular TF. Platelets also facilitate leukocyte recruitment to activated endothelium by forming P-selectin-PSGL-1 mediated conjugates with circulating leukocytes [7].

The many functions of TF and thrombin also are good illustrations of the extensive cross-talking between inflammation and coagulation. Inflammatory cytokines induce TF expression in leukocytes and in endothelial cells. A complex formation between TF and the coagulation factor FVIIa or Xa is instrumental in initiating coagulation on negatively-charged cell membranes, whereas membrane-bound TF is also capable of signal transduction directly, mediating inflammatory reactions [8]. Also, the major coagulation enzyme thrombin has many functions beyond haemostasis. It is a key regulator of cellular activation by activating PAR-receptors present on platelets but also on endothelial cells, thus triggering inflammatory pathways [9]. Of note, the generation of thrombin during hemostatic activation extends well beyond the moment at which coagulation occurs, leaving room for thrombin-mediated activity beyond hemostasis.

Fibrin also mediates cellular activation. It recruits and activates platelets through the platelet receptor α_IIb_β_3_, but also leukocytes through interaction with the α_M_β_2 _(mac-1) integrin receptor [10]. Leukocyte binding to fibrin profoundly alters leukocyte function, leading to phagocytosis, NFκB-mediated transcription, production of chemokines and cytokines and degranulation.

The two-way relationship between inflammation and coagulation is apparent in the procoagulant pathways, but also in the natural anticoagulant pathways that all have been attributed anti-inflammatory properties. The three major natural anticoagulants (Tissue Factor Pathway Inhibitor (TFPI), Antithrombin (AT), Protein C/S) have been investigated in clinical trials in patients with severe sepsis, with varying success. The anti-inflammatory properties of thrombomodulin are increasingly recognized [11]. Thrombin, bound to endothelial thrombomodulin, activates Protein C, which together with its cofactor protein S decelerates the coagulation cascade by inactivating factors Va and VIIIa. This ingenious mechanism also exerts anti-inflammatory properties. Even when the molecular dynamics of APC-mediated PAR-1 activation are incompletely understood and subject to controversial interpretation, the importance of the protein C system in damping inflammation was confirmed in the PROWESS trial [12]. In this randomized, controlled trial, the administration of recombinant human activated protein C (drotrecogin-alfa) decreased mortality in patients with severe sepsis, a condition characterized by massive activation of the coagulation system and vascular inflammation.

Endothelial cells integrate pro- and anticoagulant pathways but also pro- and anti-inflammatory stimuli. Endothelial cells are actively involved in haemostasis, limiting clot formation to sites of injury, but also localizing inflammatory processes to areas of damage, in part via common pathways. Frequently used denominators as endothelial "activation" or "stimulation" do not cover the complexity of the endothelial cell response to different physiological and pathological stimuli such as bacterial invasion (sepsis, endocarditis) and exposure to toxins (Shigella, uremic toxins), trauma (including lung transepithelial passage of pollutants) or cytokines (tissue inflammation, tumor burden). Inflammation shifts endothelial cells towards a more prothrombotic state, downregulating natural anticoagulant defense mechanisms and leading to expression and *de novo *synthesis of prothrombogenic molecules such as P-selectin, von Willebrand factor and possibly also TF. Activated or apoptotic endothelial cells may also release TF microvesicles which can contribute to the pool of circulating microvesicular TF. Nonetheless, it would be an oversimplification to state that in pathological circumstances the endothelial cells change from an anticoagulant, anti-inflammatory and vasodilatory phenotype to a procoagulant and proinflammatory state.

Coagulation and inflammation are closely linked, both in health and disease. Indeed, failure of the complex balance between pro- and anticoagulation, or between pro- and anti-inflammatory reactions because of genetic or acquired disturbances may result in disease. The many links between inflammation and coagulation help explain the prothrombotic tendency observed in patients with acute inflammatory or infectious diseases. In addition, chronic inflammatory diseases also predispose to venous thrombosis but may also accelerate atherogenesis - a low-grade vascular inflammatory disease. The endothelium serves as the interface between inflammation and inappropriate activation of the coagulation system in diffuse intravascular coagulation and thrombotic microangiopathies. Polymorphisms may predispose to disease severity. A common polymorphism in the PAI-1 gene has been reported to affect the likelihood of patients with a meningococcal infection to develop sepsis [13]. Mutations in both complement factors as well as in thrombomodulin have been linked to atypical hemolytic uremic syndrome [9]. Factor XIIa, for a long time considered as an outsider in the coagulation cascade, bridges the coagulation system with the kallikrein-kininogen system and the complement cascade, probably a remnant of the common ancestry of the different systems. A gain of function mutation in factor XII has been linked to angio-oedema, similar to deficiency in C1 esterase inhibitor. Many other disease-modifying polymorphisms still need to be elucidated.

Pathogens have developed many strategies to counteract the host defense system; some are of particular interest in the perspective of the cross-talk between coagulation and inflammation. The bacterial plasminogen activators are well characterized: streptokinase from *Streptococcus pyogenes*, staphylokinase from *Staphylococcus aureus *and the plasminogen activator Pla from *Yersinia pestis *proved pivotal for the spread of the bacteria in the host. The discovery of streptokinase paved the way for the current thrombolytic therapy. In order to enable feeding on mammals, the leech *hirudo medicinalis *uses anticoagulants and antiplatelet products to prevent clotting by the host. The direct thrombin inhibitor lepirudin, has been derived from the leech and was the first direct thrombin inhibitor for clinical use.

Vascular biology has evolved as a research tool to help explain a broad range of non-vascular diseases. Its role in helping to unravel the pathophysiology of inflammatory and especially infectious diseases has just started. Understanding the molecular mechanisms of these diseases offers the perspective for new therapies and enlarges the research field for researchers in vascular biology, thrombosis and haemostasis.

## Competing interests

The authors declare that they have no competing interests.

## Authors' contributions

PV and MH contributed to the concept of this Editorial and wrote the manuscript. Both authors read and approved the final manuscript.
